# A High Fiber Cookie Made with Resistant Starch Type 4 Reduces Post-Prandial Glucose and Insulin Responses in Healthy Adults

**DOI:** 10.3390/nu9030237

**Published:** 2017-03-05

**Authors:** Maria L. Stewart, J. Paul Zimmer

**Affiliations:** Global Nutrition R & D, Ingredion Incorporated, 10 Finderne Ave, Bridgewater, NJ 08807, USA; paul.zimmer@ingredion.com

**Keywords:** resistant starch type 4, dietary fiber, post-prandial, blood glucose, insulin, capillary glucose, glycemic response

## Abstract

Distarch phosphate is a resistant starch type 4 (RS4) containing phosphodiester cross-links within and between starch molecules. This study examined the glycemic effects of VERSAFIBE 1490™ resistant starch, a distarch phosphate derived from potato, containing 90% total dietary fiber (TDF, AOAC 991.43 method). In this double-blind, randomized, placebo-controlled, cross-over study, 28 healthy adults consumed a cookie containing 24 g fiber from distarch phosphate (fiber cookie) or a control cookie containing 0.5 g fiber that was matched for fat, protein, and total carbohydrate content. Intravenous blood glucose, intravenous blood insulin, and capillary glucose were measured for two hours after cookie consumption. The fiber cookie reduced the post-prandial blood glucose incremental area under the curve from 0 to 120 minutes (iAUC_0-120min_) by 44% (*p* = 0.004) and reduced the maximum glucose concentration (C_max0-120min_) by 8% (*p* = 0.001) versus the control cookie. Consumption of the fiber cookie resulted in a significant 46% reduction of the post-prandial serum insulin iAUC_0-120min_ (*p* < 0.001) and a 23% reduction in Cmax_0-120min_ (*p* = 0.007) versus the control cookie. This study shows that distarch phosphate RS4 can be incorporated into a cookie and significantly reduce post-prandial glucose and insulin responses in healthy adults.

## 1. Introduction

Resistant starch (RS) is a complex carbohydrate (glucose polymer) that resists digestion and absorption in the small intestine. Resistant starches are classified into five types: RS1 (physically inaccessible starches), RS2 (granular starches with B- or C-polymorph), RS3 (retrograded starches), RS4 (chemically modified starches), and RS5 (amylose-lipid complexes) [1]. Resistant starch type 4 is a unique class of resistant starch due to the diversity of chemical modifications that decrease digestibility. Common chemical modifications include cross-linking, substitution, and pyrodextrinization [2].

Resistant starch, in general, is known for improving physiological endpoints such as improving bowel function [3] and controlling glycemia [4]. In these reviews, all sources of RS are combined. However, it has been documented that different RSs exert different physiological effects. In a study using a porcine model, RS3 increased fecal nitrogen excretion compared to RS2 [5]. The composition of the human gut microbiota was affected in different manners, depending on the type of RS consumed (RS2 vs. RS4) [6]. Given these differences, clinical trials on specific resistant starch preparations are necessary to confirm the beneficial physiological effects.

As noted previously, RS4 includes starches that have a variety of chemical modifications to reduce digestibility. VERSAFIBE™ 1490 resistant starch is a distarch phosphate derived from potato that has been modified using phosphorus oxychloride [7]. Distarch phosphate is resistant to digestion due to the presence of diester phosphate crosslinks within and between starch molecules. Phosphated distarch phosphate is a similar type of RS4 with additional monophosphate esters, although the monophosphate esters do not substantially affect digestibility [8,9].

Resistant starch type 4, specifically distarch phosphate and phosphate distarch phosphate, is a relatively new form of resistant starch, with the earliest clinical trials on phosphated distarch phosphate published in 2010. The evidence on phosphated distarch phosphate RS4, albeit limited, supports improvement of metabolic endpoints such as reduced post-prandial glucose response and reduced serum lipids, after the ingredient is consumed [10,11,12,13]. To date, only one study examined post-prandial blood glucose response to distarch phosphate (consumed in a beverage) [14]. The aforementioned study demonstrated that distarch phosphate did not contribute to the post-prandial glycemic response, and this effect needed to be confirmed when the ingredient is incorporated into a solid, baked food with mixed nutrients. The present study assessed the acute, post-prandial glycemic and insulinemic response to a cookie containing RS4 in the form of distarch phosphate (VERSAFIBE 1490 resistant starch) in healthy adults. This is the first clinical study to examine these outcomes in a solid food containing distarch phosphate RS4.

## 2. Materials and Methods

### 2.1. Study Subjects

**Figure 1:**
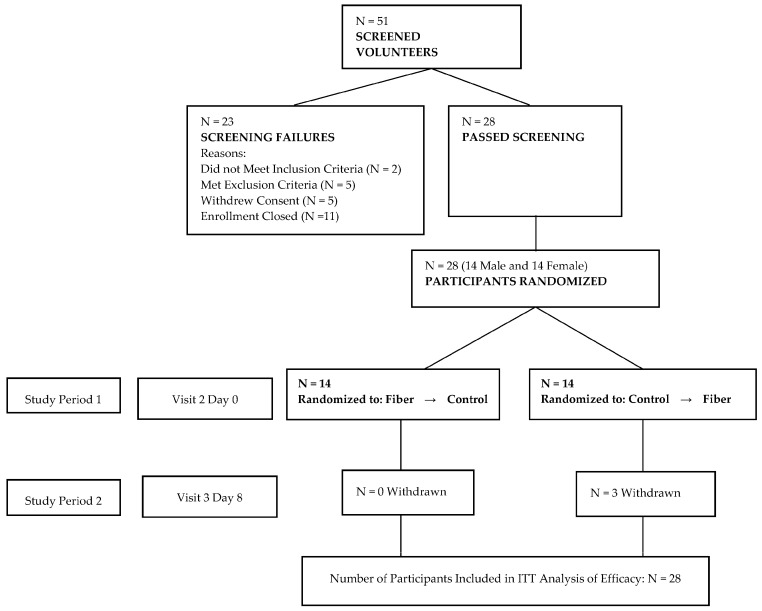
Subject flow through study.

This study was conducted in accordance with the ethical principles outlined in the Declaration of Helsinki and approved by the Institutional Review Board (IRB Services, Aurora, ON, Canada). Clinical study visits were held at a clinical research facility (KGK Synergize, London, Ontario, Canada). Healthy subjects were recruited to participate in this study. Subjects that met the inclusion criteria (18 years of age or older, body mass index (BMI) 18.0–29.9 kg/m^2^, fasting glucose ≤6.0 mmol/L; if female, not of childbearing potential (e.g., taking oral contraceptives, past hysterectomy)) and exclusion criteria (diagnosed metabolic or chronic diseases (e.g., type-2 diabetes); cancer diagnosis or treatment within 5 years; gastrointestinal problems; bowel cleansing during prior week; current medications to control blood glucose; blood cholesterol and/or blood pressure; smoker; use of medical marijuana; alcohol or drug abuse treatment in past 12 months; allergy or sensitivity to study products; blood donation in prior 2 months; if female, currently pregnant, currently breastfeeding, or planning to become pregnant) were qualified to participate in the study. Subjects provided informed consent and were randomly assigned to a treatment order at the time of enrollment: “control cookie-fiber cookie” or “fiber cookie-control cookie”. A senior staff member not involved in the study procedures generated two randomization lists—one list for males and one list for females—by www.randomization.com. Fourteen male participants were randomized into seven blocks by utilizing a randomization seed 10,087 and fourteen female participants were randomized into seven blocks by utilizing a randomization seed 11,065. A total of twenty-eight subjects were enrolled in the study. Subject flow through the study is described in Figure 1.

### 2.2. Study Design

This study was a double-blind, randomized, controlled, cross-over intervention study. Fifty-one subjects were screened, and 28 subjects were enrolled in the study (Figure 1). The subjects participated in two 24-h study periods that began the evening before the clinical study visit. Prior to each clinical study visit, the subjects consumed a standard dinner meal. Subjects arrived at the study center the following morning, after fasting for 12 h. Fasting blood samples (intravenous and capillary) were taken prior to study product consumption. Both intravenous and capillary blood samples were taken because previous reports indicated differences in blood glucose measures, depending on the sampling technique [15]. The study product (cookie) was consumed with 250 mL water. Intravenous and capillary blood samples were taken at 15, 30, 45, 60, 90, and 120 min after cookie consumption. Biochemical analyses were conducted by Life Labs (Hamilton, ON, Canada). Subjects completed a seven-day washout period between study visits.

### 2.3. Study Foods

The fiber cookie contained 25 g of VERSAFIBE™ 1490 resistant starch (Ingredion Incorporated, Bridgewater, NJ, USA), which was the primary source of fiber in the cookie. VERSAFIBE™ 1490 resistant starch is a resistant starch type 4 with 90% dietary fiber (AOAC 991.43). VERSAFIBE™ 1490 resistant starch is produced from food grade potato starch. The raw food starch is slurried in water and maintained at a temperature not exceeding 100° F. The pH of the slurry is raised not to exceed pH 12 in the presence of salt. To phosphorylate the starch, phosphorus oxychloride is added to the slurry while maintaining the reaction pH. After the phosphorylation step is complete, the pH is neutralized with acid. The starch is washed, dewatered, and dried to a moisture content not to exceed 18%.

The fiber cookie and control cookie were matched for fat, protein, and total carbohydrate (Table 1). Nutrient composition of the cookies was calculated using Genesis R&D Food Labeling Software (ESHA Research, Salem, OR, USA). The cookies were identical in appearance. The cookies were packaged in an opaque enveloped with an alpha-numeric code for identification. Neither the study subjects nor the investigators knew the identity of the cookies. The subjects rated the cookies on appearance, texture, flavor and acceptance using modified visual analog scale with demarcations at whole numbers 1–10. The subjects were not trained sensory panelists.

### 2.4. Sample Size Calculation

The sample size of 28 subjects was determined based on the primary outcome of detecting a difference in incremental area under the curve from 0 to 120 minutes (iAUC_0-120min_) intravenous blood glucose at 80% power, 0.05 alpha, and expected subject dropout rate of 12.5%.

### 2.5. Statistical Analysis

Incremental area under the curve (iAUC) was calculated using the trapezoidal approximation but only included the positive area components above the baseline value [16]. The maximum concentration (Cmax) was taken to be the highest concentration within the respective time interval. The iAUC calculations as well as the Cmax values were reported and compared for each product group.

Each numeric outcome was assessed for normality using visual representations (histogram, quantile-quantile plot, etc.) and the Shapiro–Wilk normality test. Outcomes that were log-normally or square root normally distributed were analyzed in the logarithmic or square root domain respectively. Non-normal variables were analyzed by appropriate non-parametric tests (see below). All summary statistics were reported non-transformed, arithmetic means.

Numerical efficacy endpoints were formally tested for significance between groups by a linear mixed model with a fixed effect for study product group and a random effect for each participant [17]. The concentrations of the analytes at each time point included a covariate for the baseline value. Numerical endpoints that are intractably non-normal were assessed by the Wilcoxon sign-rank test. All statistical analysis was completed using the R Statistical Software Package Version 3.2.2 (R Core Team, 2015) for Microsoft Windows. Linear mixed models were run using the “nlme” package [18]. Statistical significance was achieved at *p* < 0.05.

## 3. Results

### 3.1. Demographics

Subject demographics are shown in Table 2. All of the subjects were healthy. The demographic characteristics are consistent with the typical North American adult population.

### 3.2. Post-Prandial Blood Glucose and Insulin Response

Three subjects who were randomized to the “control cookie-fiber cookie” sequence did not complete the second treatment. Blood samples obtained for up to two subjects in each treatment group could not be analyzed for the metabolite of interest. The sample size for each group and each outcome is noted in the footnote of Table 3.

**Figure 2:**
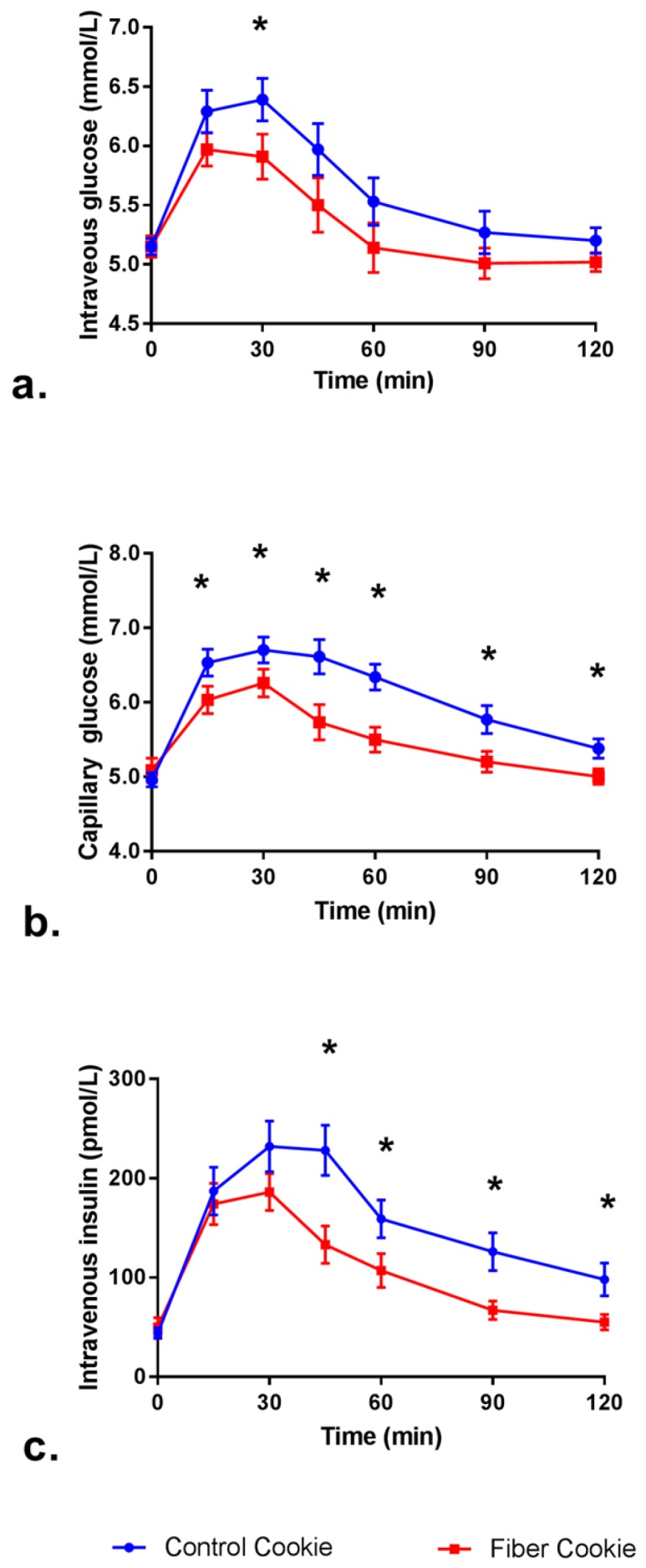
Post-prandial blood measurements over 120 min (**a**) intravenous blood glucose; (**b**) capillary blood glucose; (**c**) intravenous insulin concentrations. Data are presented as means ± SEM. * = Fiber cookie and control cookie were significantly different (*p* < 0.05).

Mean post-prandial intravenous blood glucose, capillary glucose, and intravenous serum insulin concentration over the two-hour study period are shown in the time-course graphs (Figure 2A–C). Intravenous blood glucose was significantly lower at 45 min after the fiber cookie was consumed, compared to the control cookie. Capillary blood glucose concentrations were significantly lower at 15, 30, 45, 60, 90, and 120 min after the fiber cookie was consumed, compared to the control cookie. At 45, 60, 90, and 120 min, intravenous blood insulin concentrations were significantly lower after subjects consumed the fiber cookie compared to the control cookie.

The significant reductions at individual time points for glucose and insulin values reflected significant reductions in iAUC and Cmax. After consuming the fiber cookie, the subjects experienced a 44% reduction in intravenous blood glucose iAUC_0-120min_ compared to the control cookie (*p* = 0.004, Table 3). This was largely driven by a significant, 8% reduction in intravenous blood glucose Cmax_0-120min_ after consuming the fiber cookie compared to the control cookie (*p* = 0.001). A similar response was noted for capillary blood glucose measures, with a significant, 48% reduction in iAUC_0-120min_ and a significant 9% reduction in Cmax_0-120min_. Intravenous blood insulin was significantly lower for iAUC_0-120min_ (46% lower), and Cmax_0-120min_ (23% lower), after subjects consumed the fiber cookie, compared to the control. The decrease in insulin concentrations after consuming the fiber cookie reflect the decreased intravenous and capillary blood glucose concentrations.

The subject ratings were favorable and did not differ between groups (Table 4).

## 4. Discussion

Dietary fiber has been long acknowledged for reducing post-prandial blood glucose and insulin concentrations through mechanisms of delayed nutrient absorption or replacement of digestible carbohydrates [19]. The RS4 used in this trial, VERSAFIBE 1490 resistant starch, replaced digestible carbohydrates from refined flour when formulated into processed foods such as bakery items. This fiber maintains sensory attributes of the final food while increasing dietary fiber and decreasing available carbohydrate content. Resistant starch type 4 is a broad class of resistant starches that have been chemically modified to reduce digestibility. The particular ingredient used in this study contains phosphodiester cross-links in the distarch phosphate molecules that reduce swelling and enzyme accessibility [8,9,20]. Previous work demonstrated the low glycemic response to distarch phosphate when mixed with water alone [14]. When the resistant starch type 4 was added to a dextrose beverage, the glycemic response was the same as the dextrose beverage alone. This demonstrates that the RS4 does not affect bioavailability of other carbohydrates, and the changes in post-prandial glycemic response are due to the nondigestible nature of the carbohydrate. The dietary fiber content is consistent when analyzed with both AOAC method 991.43 and AOAC method 2009.01, which indicates that the RS4 is heat-stable and resistant to prolong enzymatic digestion (unpublished data). We expect the RS4 content and dietary fiber content to be similar in the ingredient as well as the final food product (cookie).

Resistant starch type 4 may exert additional mechanisms to reduce post-prandial glycemic response, in addition to strictly replacing available carbohydrate. In a study where the treatments were matched for available carbohydrate, the RS4 treatment, phosphated distarch phosphate, resulted in significantly lower blood glucose iAUC_0-120min_, peak blood glucose, blood insulin iAUC_0-120min_ and peak insulin in healthy adults [10]. Additional research is needed to further define the mechanisms by which RS4 lowers post-prandial blood glucose response when treatments are matched for available carbohydrate.

The iAUC_0-120min_ and Cmax_0-120min_ for blood glucose were significantly lower after subjects consumed the fiber cookie compared to the control cookie, regardless of the sampling method. As noted by previous researchers, the absolute blood glucose values differ when measured intravenously or through capillary sampling [15]. When individual time points were compared, the capillary sampling method yielded significantly lower values after the fiber cookie was consumed at 45, 60, 90, and 120 min, whereas the intravenous sampling method yielded significantly different blood glucose values at 45 min, only. This can be attributed to the larger variability in blood glucose values when intravenous sampling was used. Previous researchers also noted this phenomenon [15]. Intravenous insulin iAUC_0-120min_ and Cmax were lower after the subjects consumed the fiber cookie compared to the control cookie, which corresponds to the observed changes in blood glucose.

Resistant starch type 4, such as distarch phosphate, has functional properties that allow it to replace refined grain flour in product formulations. As a result, the available carbohydrates in a food can be reduced while maintaining the same sensory properties [2]. This provides the opportunity to formulate desirable foods with added health benefits such as improved post-prandial blood glucose management.

Reduced post-prandial glycemic response is a beneficial health effect for healthy individuals as well as individuals with compromised carbohydrate metabolism (e.g., pre-diabetes, Type-2 diabetes). The results from this study demonstrate how replacing refined flour with RS4 in a baked good (cookie) reduces post-prandial glucose and insulin response in healthy adults. Further research is warranted in individuals with compromised carbohydrate metabolism.

## Figures and Tables

**Table 1:** Nutrient composition of cookies.

Per Serving, As-Eaten	Control Cookie	Fiber Cookie
Weight (g)	47.02	48.00
Calories (kcal)	214.7	129.7
Fat (g)	3.99	3.92
Saturated fat (g)	0.56	0.54
Protein (g)	5.36	4.92
Total Carbohydrates (g)	36.84	36.84
Available Carbohydrates (g)	36.28	12.71
Dietary Fiber (g) *	0.55	24.13
Sugars (g)	11.51	11.72

* Fiber cookie contained VERSAFIBE 1490 resistant starch.

**Table 2:** Subject demographics.

Mean ± SD	All Participants (*n* = 28)
Age (y)	42.8 ± 18.5
Sex (male/female)	14/14
Race (white/nonwhite)	23/5
Weight (kg)	71.3 ± 12.0
Body Mass Index (kg/m^2^)	24.7 ± 3.3
Fasting blood glucose (mmol/L)	5.03 ± 0.34

**Table 3:** Post-prandial glucose and insulin iAUC and Cmax **^§^**.

Mean ± SD	Control Cookie	Fiber Cookie	*p*-Value ^§^
Intravenous blood glucose *			
iAUC_0-120min_ (mmol/L *·h)	1.31 ± 0.75	0.73 ± 0.90	0.004
Cmax_0-120min_ (mmol/L *·h)	6.83 ± 0.90	6.29 ± 0.82	0.001
**Capillary blood glucose ^†^**			
iAUC_0-120min_ (mmol/L *·h)	2.35 ± 0.94	1.22 ± 1.18	<0.001
Cmax_0-120min_ (mmol/L *·h)	7.22 ± 1.00	6.60 ± 1.00	0.005
**Intravenous serum insulin ^‡^**			
iAUC_0-120min_ (pmol/L *·h)	229 ± 124	124 ± 94	<0.001
Cmax_0-120min_ (pmol/L *·h)	280 ± 129	215 ± 94	0.007

**^§^** iAUC = incremental area under the curve, Cmax = maximum concentration * *n* = 27 control cookie, *n* = 25 fiber cookie; ^†^ *n* = 26 control cookie, *n* = 23 fiber cookie; ^‡^ *n* = 27 control cookie, *n* = 25 fiber cookie; **^§^** iAUC intravenous blood glucose and iAUC intravenous serum insulin datasets were square root transformed prior to statistical analysis; Cmax intravenous blood glucose and Cmax intravenous serum insulin datasets were log transformed prior to statistical analysis.

**Table 4:** Sensory ratings of the fiber cookie and control cookie.

Question	Fiber Cookie	Control Cookie	Between Group *p* Value ^§^
	Mean ± SD (*n*)	Mean ± SD (*n*)	
Rate the appearance of the cookie	5.69 ± 2.02 (26)	5.61 ± 1.89 (28)	0.811
Rate the texture of the cookie	6.23 ± 2.07 (26)	5.93 ± 2.07 (28)	0.525
Rate the flavor of the cookie	6.46 ± 2.14 (26)	6.79 ± 1.62 (28)	0.355
What is your overall acceptance of the cookie?	6.54 ± 2.21 (26)	6.50 ± 1.58 (28)	0.970

^§^ Between group comparisons were made using RM-ANOVA not adjusted for baseline. Probability values *p* ≤ 0.05 are statistically significant.
